# Impact of Helminth Infection during Pregnancy on Cognitive and Motor Functions of One-Year-Old Children

**DOI:** 10.1371/journal.pntd.0003463

**Published:** 2015-03-10

**Authors:** Michael O. Mireku, Michael J. Boivin, Leslie L. Davidson, Smaïla Ouédraogo, Ghislain K. Koura, Maroufou J. Alao, Achille Massougbodji, Michel Cot, Florence Bodeau-Livinec

**Affiliations:** 1 Université Pierre et Marie Curie (UPMC- Paris VI), Paris, France; 2 Ecole des Hautes Etudes en Santé Publique, Département d’Épidémiologie et des Biostatistiques, Rennes, France; 3 Institut de Recherche pour le Développement (IRD), Mère et Enfant face aux Infections Tropicales, Paris, France; 4 Michigan State University, Departments of Psychiatry and Neurology/Ophthalmology, East Lansing, Michigan, United States of America; 5 Columbia University, Mailman School of Public Health and the College of Physicians and Surgeons, New York, New York, United States of America; 6 Centre Hospitalier Universitaire Yalgado Ouédraogo, Ouagadougou, Burkina Faso; 7 Union Internationale Contre la Tuberculose et les Maladies Respiratoires, Département Tuberculose et VIH, Paris, France; 8 Hôpital de la Mère et de l’Enfant Lagune de Cotonou, Service de Pédiatrie, Cotonou, Bénin; 9 Université d’Abomey-Calavi, Faculté des Sciences de la Santé, Cotonou, Bénin; 10 PRES Sorbonne Paris Cité, Université Paris Descartes, Faculté des Sciences Pharmaceutiques et Biologiques, Paris, France; 11 Inserm UMR 1153, Obstetrical, Perinatal and Pediatric Epidemiology Research Team (Epopé), Center for Epidemiology and Statistics Sorbonne Paris Cité, DHU Risks in Pregnancy, Paris Descartes University, Paris, France; 12 New York University Medical Center, Division of Parasitology, Department of Microbiology, New York, New York, United States of America; Christian Medical College, INDIA

## Abstract

**Objective:**

To determine the effect of helminth infection during pregnancy on the cognitive and motor functions of one-year-old children.

**Methods:**

Six hundred and thirty five singletons born to pregnant women enrolled before 29 weeks of gestation in a trial comparing two intermittent preventive treatments for malaria were assessed for cognitive and motor functions using the Mullen Scales of Early Learning, in the TOVI study, at twelve months of age in the district of Allada in Benin. Stool samples of pregnant women were collected at recruitment, second antenatal care (ANC) visit (at least one month after recruitment) and just before delivery, and were tested for helminths using the Kato-Katz technique. All pregnant women were administered a total of 600 mg of mebendazole (100 mg two times daily for 3 days) to be taken after the first ANC visit. The intake was not directly observed.

**Results:**

Prevalence of helminth infection was 11.5%, 7.5% and 3.0% at first ANC visit, second ANC visit and at delivery, respectively. Children of mothers who were infected with hookworms at the first ANC visit had 4.9 (95% CI: 1.3–8.6) lower mean gross motor scores compared to those whose mothers were not infected with hookworms at the first ANC visit, in the adjusted model. Helminth infection at least once during pregnancy was associated with infant cognitive and gross motor functions after adjusting for maternal education, gravidity, child sex, family possessions, and quality of the home stimulation.

**Conclusion:**

Helminth infection during pregnancy is associated with poor cognitive and gross motor outcomes in infants. Measures to prevent helminth infection during pregnancy should be reinforced.

## Author Summary

The WHO recommends anthelmintics for pregnant women after their first trimester but the benefits remain unequivocal. Although the consequences of helminth infection during pregnancy on the health of pregnant women have been well studied, the impact on the early development of offspring has been understudied. Studies suggest that helminth infection in children may be associated with poor cognitive development, but very little is known whether a similar consequence exists for offspring of women infected with helminths during pregnancy. From our study, we found that women who had intestinal worm infection at least once during pregnancy, had children with lower cognitive and motor scores. Moreover, hookworm infection in pregnant women prior to receiving anthelminthic treatment was associated with poor gross motor functions of children at one-year of age. Based on the findings of this study, measures to prevent helminth infections during pregnancy should be reinforced.

## Introduction

Intestinal helminths infect more than two billion of the world’s population, with the highest prevalence in Asia and sub-Saharan Africa.[1] The burden of intestinal helminth infection is estimated to be five million disability-adjusted life years (DALYs).[2] Helminth infections are rarely directly associated with increased mortality but are related to increased morbidity arising from the chronicity and consequences of infection.[3] Although the World Health Organization (WHO) highly recommends anthelmintic therapy for pregnant women in their second trimester[4], the benefits on anemia, congenital anomalies and perinatal mortality remains unequivocal[5]. In sub-Saharan Africa, it is estimated that one-third of pregnant women are infected with soil-transmitted helminths[6] although several studies have shown wide variation in prevalence across different countries, 11.1% in Benin[7], 25.7% in Ghana[8] and 49% in Gabon[9]. In Benin, anthelminthics are a component of the routine antenatal care (ANC) package given to pregnant women after their first trimester.[10]

A recent systematic review found little evidence that deworming in children is associated with better cognitive function, though most trials included were of poor quality.[11] A cross-sectional study revealed that compared to 7 to 18 year-old-children who were not infected with *Ascaris lumbricoides* and *Trichuris trichiura*, children who were infected with either of these species of helminth performed poorly on tests of memory and verbal fluency, respectively.[12] Over the past decades, many studies have confirmed helminth infection during pregnancy as a risk factor for maternal iron deficiency (ID) and anemia[3,13,14]. However, evidence remains limited on the effects on adverse birth outcomes such as low birth weight (LBW) [15] which is known to be associated with poorer cognitive function in children.[16] Additionally, ID and anemia during pregnancy may be associated with poor cognitive function of infants as shown in a study in rural China which revealed that children of iron deficiency anemic (IDA) women performed significantly lower than those of non-IDA women in cognitive assessment tests.[17] The rapid rate of development of fetal organs makes them particularly susceptible to prenatal insults that are injurious to fetal development, and which could influence their development persisting even after birth. The early onset of delayed cognitive development could negatively influence several aspects of child development including preparedness for school.[18] Notwithstanding the evidence that helminths are associated with these indirect threats, very little is known about the impact of helminth infection during pregnancy on actual infant cognitive development. A study in Uganda concluded that *Mansonella perstans* and *Strongyloides stercoralis* infection during pregnancy may be associated with impaired executive function in children.[19]

The objective of this study was to determine whether maternal infection with helminths, both in general and with specific helminth species, during pregnancy, is associated with cognitive and gross motor functions of one-year-old children in Benin.

## Methods

### Population

Our prospective cohort included singletons born to pregnant women who were enrolled before 29 weeks of gestation in the *Malaria in Pregnancy Preventive Alternative Drugs* (*MiPPAD*) clinical trial (NCT00811421) comparing sulfadoxine-pyrimethamine and mefloquine as intermittent preventive treatment of malaria in pregnancy (IPTp). The study was conducted in the district of Allada in Benin. One thousand and five HIV-negative pregnant women attending their first ANC visit in the health centers in each of the three sub districts of Allada (Sekou, Allada and Attogon) were recruited. Detailed inclusion and exclusion criteria in the *MiPPAD* trial are explained elsewhere.[7]

All live born children of recruited pregnant women who survived to 12 months were invited for neurocognitive assessment in the TOVI study (Fon language: *Tovi* means Child from the country).

### Variables

#### Pregnant women

Socio-demographic and clinical data were collected at three ANC visits; at recruitment (first ANC visit), during the 2^nd^ dose of IPTp (second ANC visit, at least one month after recruitment) and at delivery. At first ANC visit, information on gravidity, gestational age, anthropometric data and socio-demographic characteristics including age, education, and occupation were collected. Details of biological assessments for pregnant women and how prepregnancy BMI was calculated have been described in an earlier publication.[7] More specifically, data on maternal helminth infection comes from a parallel cohort study (Anemia in Pregnancy: Etiologies and Consequences “APEC”) which was nested within the MiPPAD trial.[20] At ANC visits, women were given IPTp, iron and folic acid as part of the ANC package in Benin. Women were also given a total of 600 mg of mebendazole (100 mg twice daily for 3 days) at the first ANC visit and again if they were tested positive for helminths at second ANC visit. Intake of mebendazole was not directly monitored. Containers were given to pregnant women to collect stool samples from the next morning after the ANC visits. Since it was difficult to obtain stool samples when women were in labor, where possible, stool samples were collected 15 days prior to the expected date of delivery or within a week after delivery.

Thick smears of retrieved stool samples were immediately prepared and assessed using the Kato-Katz technique as described by the WHO[21]. One of the two slides prepared from each sample was systematically and independently examined by two laboratory technicians under a microscope. The mean of the two results was calculated and reported. To obtain the standardized fecal egg counts (FEC), in eggs per gram of stool (epg), for each individual stool sample, the counted number of eggs for each species of helminth was multiplied by twenty-four. *T*. *trichiura, A*. *lumbricoides* and *S*. *mansoni* were not independently considered as specific exposures in the univariate and multivariate analyses owing to their low prevalence throughout pregnancy. Also, due to the low intensity of hookworms in our study population, the WHO cut-off for classification[22] was not applicable. Instead, the intensity of helminth infection was categorized using the median as a cut-off value. In this article, helminth infection is defined as the presence of at least one egg of any species of helminth in the stool.

Gestational age was assessed according to fundal height at delivery.

Within three days after the cognitive assessments of children at age one year, a different nurse conducted home visits during which information on family possessions was collected and the Raven’s Progressive Matrices (RPM) test was conducted. A score was generated for family possessions, which was the sum of individual scores given to specific items owned by the family of the pregnant woman thus whether the home had electricity or car (each scoring 3), a motorcycle or television (each scoring 2) and a radio, bicycle, motorcycle, or cattle (each scoring 1). The RPM uses an approach which does not require verbal proficiency to provide an estimate of intelligence quotient[23].

#### Infants

As part of the APEC study, stool samples were collected from a subsample of children (N = 186) at 6, 9 or 12 months for examination for helminths. At the health centers, trained research nurses individually assessed the cognitive function of 635 one-year-old infants using the Mullen Scales of Early Learning (MSEL) from April 2011 to November 2012. The MSEL which comprises of five scales, gross motor, fine motor, visual reception, receptive language and expressive language, was adapted to this setting prior to its use in this study.[24]

From the raw scores obtained by infants in each MSEL scale, normalized age specific (monthly) scores called the *t*-scores were generated. *T*-scores of the visual reception, fine motor, receptive language and expressive language scales were then combined to form the Early Learning Composite (ELC) score, which is indicative of early cognitive function.[25] Detailed quality assurance and reliability of assessment have already been published.[24] Briefly, five assessors were trained by MJB and FBL in the field and retrained three times. Difficulties to be discussed with FBL and MJB were reviewed during weekly meetings. Inter-rater reliability was checked.

The Home Observatory Measurement of the Environment (HOME) inventory was administered three days after MSEL assessment during the home visit. The HOME inventory, adapted for this setting, was used to assess the quality of the home environment including the parent-child interaction and the learning opportunities available to the child at home.[26] After training and pilotting, changes were made to six items in the HOME inventory to suit our study setting. For example, one item regarding the child being outside of the house was removed as children spend most of the day outside. Description of how the HOME inventory was administered has been detailed in a previous publication. [24]

### Statistical analyses

We first described and compared the baseline characteristics of women with singleton live births whose children were assessed and those whose children were not assessed for cognitive function. Secondly, we performed univariate analyses to assess crude associations between the ELC and the gross motor scores with helminth infection, helminth species, helminth density, co-infection with malaria, and covariates [maternal prepregnancy body mass index (BMI), family possessions, maternal occupation, education, the RPM and HOME scores]. These covariates were considered as potential confounding factors as they are known risk factors for poor cognitive development and may share common causes with helminth infection. Next, we conducted a multiple linear regression adjusting for covariates whose p-values were less than 0.20 in the univariate analysis. Finally, we performed stepwise removal of covariates from the model if they were found not be statistically significant. From the final model, we evaluated the adjusted mean difference in ELC and gross motor scores. Infant characteristics at birth or age one-year including birth weight, preterm birth and infant helminth infection were hypothesized to be within the causal pathway (as mediators). All multivariate models were adjusted for infant sex. Although infant characteristics (preterm births, low birth weight, and weight-for-age at MSEL assessment) were hypothesized to be mediators in the association between prenatal helminth infection and infant cognitive function, we adjusted for these variables in a sensitivity analyses.

Statistical analyses were conducted using Stata IC/11.2 for Windows (StataCorp Lp, College station, TX). We used Pearson’s correlation to assess the associations between the dependent variables and other continuous variables. The student *t*-test, Wilcoxon rank sum test and chi-squared test were used to compare means, medians and proportions, respectively. Statistical significance was defined as p-value less than 0.05.

### Ethical considerations

The study was approved by the institutional review boards of the University of Abomey-Calavi in Benin, New York University and Michigan State University in USA and the Research Institute for Development’s (IRD) Consultative Ethics Committee in France. At recruitment, we obtained written informed consent from all pregnant women and guardians of children who participated in this study in the presence of a witness. Women who could not read and write provided thumbprints to confirm their agreement to participate in the study after a nurse had explained the study.

## Results

### General characteristics of participants

**Fig 1:**
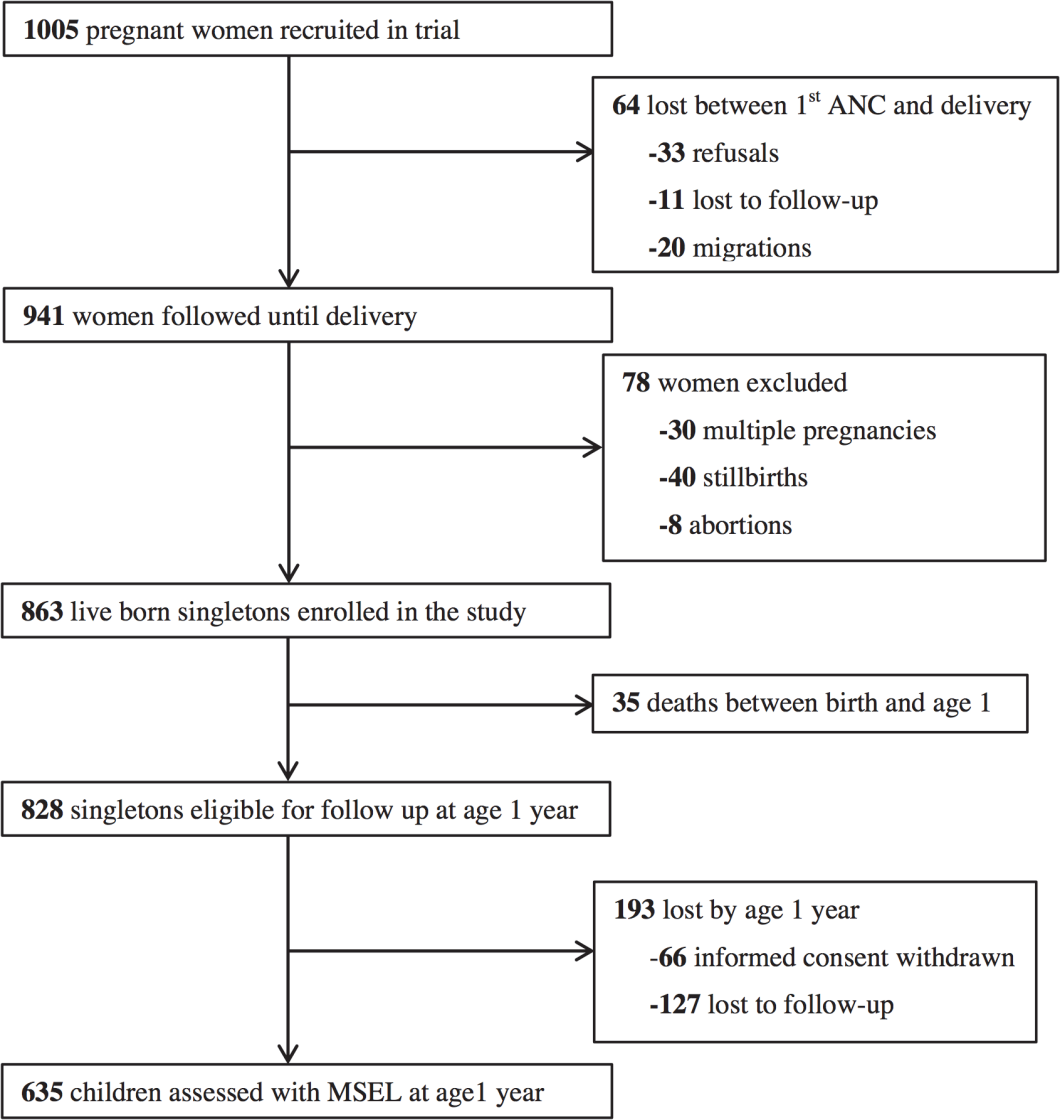
Flowchart outlining the follow-up of participating pregnant women until their children were one year old. The rate of follow-up of eligible children from birth till age one was 76.7%.

As shown in Fig. 1, 863 live born singletons were enrolled into the birth cohort but 35 died before the age of one year leaving 828 eligible children. Of these, 635 (76.7%) were assessed for cognition using MSEL at approximately one year of age. The median age during MSEL assessments was 12.1 months (range: 11.3–15.3 months). Two children were not able to complete all of the MSEL subtests, leaving 633 children who were fully assessed. Maternal baseline characteristics were similar between women whose children were fully assessed for cognitive function and those whose children were not, as shown in Table 1. Also there was no significant difference between infant characteristics between children assessed and those not assessed.

**Table 1:** Comparison of maternal baseline characteristics at first ANC visit and infant characteristics between children fully assessed and those not fully assessed for cognitive function.

Characteristics	Cognitive assessment status		*P*-value
	Fully assessed	Not/partially assessed	
	(N = 633)	(N = 230)	
**Mothers**			
Age in years [median (range)]	25 (17–45)	25 (13–42)	0.472
Gestational age in weeks [median (range)]	22 (10–28)	23 (8–28)	0.190
Gravidity			
Primigravidae	117 (18.5)	38 (16.5)	0.507
Multigravidae	516(81.5)	192 (83.5)	
Education			
Never schooled	418 (66.0)	156 (67.8)	0.622
Primary or more	215 (34.0)	74 (32.2)	
Occupation			
Housewives	313 (49.4)	107 (46.5)	0.447
Employed	320 (50.6)	125 (53.5)	
Prepregnancy BMI (kg/m²)			
Underweight (<18.5)	111 (17.5)	33 (14.4)	0.355
Normal (18.5–24.9)	456 (72.0)	167 (72.6)	
Overweight/Obese (≥25.0)	66 (10.4)	30 (13.0)	
Malaria			
Positive	101 (15.8)	31 (13.8)	0.371
Negative	532 (84.2)	199 (86.2)	
**Infants**			
Sex			
Male	310 (49.0)	104 (45.2)	0.329
Female	323 (51.0)	126 (54.8)	
Birth weight (kg)^a^			
Low (<2.5)	56 (9.5)	28 (13.3)	0.124
Normal (≥2.5)	531 (90.5)	182 (86.7)	
Gestational age at birth (weeks)^b^			
Preterm (<37)	43 (7.0)	17 (8.0)	0.631
Not Preterm (≥37)	572 (93.0)	196 (92.0)	
Weight at 1 year (kg)^c^	8.4	—	NA

Figures are number (percentage) unless otherwise indicated

^a^N = 797

^b^N = 828

^c^N = 626

BMI-Body Mass Index

### Helminth infection among pregnant women and among children

At first ANC visit, the prevalence of helminth infection was 11.5% of which hookworm infections were the most prevalent (9.5%). Of the 52 women with hookworm infections at the second ANC visit, 12 were infected with the same species at first ANC (see Table 2 for prevalence and density of helminths). The prevalence (95% CI) of helminth infection among children by age one was 32.8% (26.0%-39.6%).

**Table 2:** Descriptive assessment of helminth infection in enrolled mother- infants pairs from Benin with follow-up from 29 weeks gestation through child's 1st year of life.

Characteristics	Infected N (%) or median (range)
Helminth infection	
At 1^st^ ANC visit	98 (11.5)
During 2^nd^ ANC visit	62 (7.5)
At delivery	21 (3.0)
Helminth species at 1^st^ ANC visit	
Hookworms	81 (9.5)
*Ascaris lumbricoides*	9 (1.1)
*Trichuris trichiura*	7 (0.8)
*Schistosoma mansoni*	2 (0.2)
Helminth species at 2^nd^ ANC visit	
Hookworms	52 (6.3)
*Ascaris lumbricoides*	7 (0.9)
*Trichuris trichiura*	5 (0.6)
*Schistosoma mansoni*	—
Helminth species at delivery	
Hookworms	19 (2.7)
*Ascaris lumbricoides*	2 (0.3)
*Trichuris trichiura*	—
*Schistosoma mansoni*	—
Helminth infection over the course of pregnancy (2 categories)	
Never infected	540 (77.8)
Infected at least once	154 (22.2)
Helminth infection over the course of pregnancy (3 categories)	
Never infected	540 (80.2)
Infected only once	108 (16.1)
Infected twice or more	25 (3.7)
Hookworm density at 1^st^ ANC visit (epg)	72 (24–2736)
Hookworm density at 2^nd^ ANC visit (epg)	72 (24–1416)
Hookworm density at delivery (epg)	120 (24–912)
Hookworm infection over the course of pregnancy (3 categories)	
Never infected	553 (82.5)
Infected only once	102 (15.2)
Infected twice or more	15 (2.2)
Helminth infection by age one year	61 (32.8)
Helminth species in Children by age one year (N = 186)	
Hookworms	8 (4.3)
*Trichuris trichiura*	39 (20.9)
*Ascaris lumbricoides*	8 (4.3)
*Schistosoma mansoni*	3 (1.6)
*Entorobius vermicularis*	3 (1.6)

epg- eggs per gram of stool

—indicates no egg found on laboratory examination

ANC- Antenatal Care

### Maternal characteristics and ELC and gross motor scores

Maternal education, occupation, family possession, RPM and HOME scores and infant weight-for-age were associated with ELC and gross motor scores. Of note, maternal malarial infection was not statistically significantly associated with ELC and gross motor scores (see Table 3). Infant ELC and gross motor scores increased with increasing prepregnancy BMI class. As shown in Table 3, children born preterm and those with low birth weight had lower ELC and gross motor scores, respectively.

**Table 3:** Association between maternal and infant socio-demographic and anthropometric characteristics and early learning composite (ELC) and gross motor scores.

Characteristics	ELC score		Gross motor score	
N = 634	Mean	*P*-value	Mean	*P*-value
**Mothers**				
Gravidity				
Primigravidae	99.8	0.323	48.2	0.021
Multigravidae	98.3		51.6	
Maternal Education				
None	96.4	<0.0001	49.4	<0.001
Some	102.9		54.1	
Maternal Occupation				
Housewives	96.3	<0.001	49.2	0.003
Employed	100.8		52.7	
Prepregnancy BMI				
Underweight	96.3	0.061	48.9	0.029
Normal	98.7		50.9	
Overweight/Obese	101.5		54.8	
Family Possession score^a^	0.1^b^	0.004	0.2^b^	<0.0001
RPM score^a^	0.1^b^	0.035	0.1^b^	0.002
HOME score^a^	0.2^b^	<0.0001	0.2^b^	<0.0001
Malaria at 1^st^ ANC				
Positive	100.4	0.162	51.2	0.362
Negative	98.2		49.8	
Malaria at 2^nd^ ANC				
Positive	100.7	0.465	56.1	0.086
Negative	98.5		50.9	
**Infants**				
Sex				
Male	97.9	0.227	51.9	0.101
Female	99.3		50.1	
Birth weight (kg)^b^				
Low (<2.5)	97.6	0.630	45.3	0.002
Normal (≥2.5)	98.5		51.7	
Gestational age at birth (weeks)^c^				
Preterm (<37)	95.9	0.007	51.0	0.951
Not Preterm (≥37)	99.5		51.1	
Weight for age at 1 year (kg)^d^	0.1^b^	<0.001	0.2^b^	<0.0001
Helminth infection by 1 year^e^				
Positive	99.4	0.539	52.8	0.337
Negative	100.8		50.6	

ANC- Antenatal Care

BMI-Body Mass Index

^a^ N = 629

^b^N = 588

^c^ N = 594

^d^N = 627

^e^N = 182

^b^ Represented as Correlation coefficients not means

### Socio-demographic characteristics and maternal helminth infection

As shown in Table 4, maternal occupation and educational status were associated with helminth infection at second ANC visits. Family possessions score was associated with helminth infection at both ANC visits.

**Table 4:** Association between helminth infection at ANC visits and maternal and infant characteristics.

	Helminth infection at 1^st^ ANC		Helminth infection at 2^nd^ ANC	
	N (%)	*P*-value	N (%)	*P*-value
Gravidity				
Primigravidae	20 (13.2)	0.484	11 (7.2)	0.889
Multigravidae	78 (11.2)		51 (7.6)	
Maternal Education				
Never schooled	72 (12.8)	0.109	51 (9.3)	0.005
Primary or more	26 (9.1)		11 (3.9)	
Maternal Occupation				
Housewives	53 (12.8)	0.242	40 (10.0)	0.009
Employed	45 (10.3)		22 (5.2)	
Prepregnancy BMI				
Underweight	20 (14.2)	0.498	14 (3.0)	0.156
Normal	69 (11.2)		45 (7.6)	
Overweight/Obese	9 (9.6)		3 (3.2)	
HOME score (median) ^a^	27 (27)	0.642	27 (27)	0.411
Raven score (median) ^a^	15 (15)	0.733	15 (15)	0.937
Family Possession (median) ^a^	5 (5)	0.044	4 (5)	0.021
Malaria at 1st ANC visit				
Positive	18 (13.9)	0.366	12 (9.5)	0.366
Negative	80 (11.1)		50 (7.2)	
Malaria at 2nd ANC visit				
Positive	5 (15.2)	0.576	3 (8.8)	0.736
Negative	92 (11.6)		59 (7.5)	
Infant helminth infection				
At least once	5 (8.5)	0.336	6 (9.8)	0.573
Never	6 (4.8)		9 (7.3)	

BMI-Body Mass Index

^a^ Represented as median scores for women with helminth infection (median scores for uninfected women)

### Prenatal helminth infection and infant cognitive and gross motor functions

The difference in mean ELC scores between children whose mothers were infected with helminths at first ANC visit and those whose mothers were not infected with any helminth remained significant after adjusting for maternal education, child sex and HOME score (p = 0.013). Pregnant women who were infected with helminths at least, once during pregnancy had children with poorer ELC scores, thus-4.4 (95% CI: -7.2 to-1.5) compared to those of mothers who were never infected during pregnancy after adjustment (see Table 5).

**Table 5:** Relationship between helminth infection during pregnancy and mean scores of infant cognitive and gross motor function at age 1 year.

	Mean difference in ELC scores			Mean difference in gross motor scores	
	Crude beta [95%CI]	Adjusted beta[95%CI]^a^	Adjusted beta[95%CI]^b^	Adjusted beta [95%CI]^c^	Adjusted beta [95%CI]^d^
Helminth infection					
At 1st ANC visit	-4.2 [-7.6, -0.7]*	-4.3 [-7.6, -0.9]*	-4.3 [-7.7, -1.0]*	-3.9 [-7.3, -0.4]*	-4.4 [-7.9, -1.0]*
At 2nd ANC visit	-5.1 [-9.0, -1.1]*	-3.9 [-7.8, -0.0]*	-3.5 [-7.4, 0.3]	-2.2 [-6.1, 1.7]	-1.6 [-5.5, 2.4]
At delivery	-1.9 [-9.4, 5.6]	-0.2 [-7.5, 7.1]	-0.0 [-7.4, 7.4]	5.3 [-2.0, 12.6]	5.2 [-2.2, 12.9]
Hookworm					
At 1st ANC visit	-3.3[-7.1, 0.5]	-3.7 [-7.3, 0.0]	-4.0 [-7.7, -0.3]*	-4.9 [-8.6, -1.1]*	-5.5 [-9.3, -1.8]**
At 2nd ANC visit	-6.0 [-10.3, -1.6]**	-4.7 [-8.9, -0.5]*	-4.4 [-8.6, -0.2]*	-2.7 [-7.0, 1.5]	-2.2 [-6.5, 2.1]
At delivery	-1.1 [-8.9, 6.6]	0.6 [-7.0, 8.1]	1.1 [-6.6, 8.8]	6.9 [-0.7, 14.5]	7.2 [-0.6, 15.1]
Hookworm density at 1st ANC visit					
Not infected (ref)	0	0	0	0	0
Moderate (≤72, median)	-2.5 [-7.4, 2.5]	-3.2 [-8.1, 1.6]	-3.5 [-8.4, 1.4]	-5.4 [-10.3, -0.5]*	-6.5 [-11.4, -1.5]*
High (>72)	-4.4 [-9.9, 1.1]	-4.2 [-9.5, 1.2]	-4.6 [-9.9, 0.6]	-4.1 [-9.6, 1.2]	-4.4 [-9.7, 0.9]
Hookworm density at 2nd ANC visit					
Not infected (ref)	0	0	0	0	0
Moderate (≤72, median)	-5.2 [-10.4, 0.1]	-3.9 [-9.0, 1.2]	-3.7 [-8.9, 1.4]	-3.1 [-8.2, 2.0]	-2.6 [-7.8, 2.7]
High (>72)	-7.4 [-14.6, -0.2]*	-6.2 [-13.2, 0.9]	-5.7 [-12.9, 1.4]	-2.0 [-9.2, 5.2]	-2.1 [-9.3, 5.1]
Hookworm density at delivery					
Not infected (ref)	0	0	0	0	0
Moderate (≤120, median)	-2.8 [-13.3, 7.8]	-0.6 [-10.9, 9.6]	0.1 [-10.0, 10.2]	6.7 [-3.5, 17.0]	8.2 [-2.1, 18.5]
High (>120)	0.8 [-10.6, 12.1]	2.0 [-9.0, 13.0]	2.5 [-9.3, 14.4]	7.1 [-3.9, 18.2]	5.9 [-6.1, 18.0]
Occurrence of helminth infection over the course of pregnancy					
Never infected (ref)	0	0	0	0	0
At least once	-4.9 [-7.8, -2.1]***	-4.4 [-7.2, -1.5]**	-4.1 [-7.0, -1.3]**	-2.9 [-5.7, -0.0]*	-2.8 [-5.7, 0.1]
Malaria-helminth co-infection					
At 1st ANC visit	-4.0 [-12.1, 4.1]	-3.1 [-11.1, 4.9]	-2.2 [-10.1, 5.7]	-3.1 [-11.1, 4.9]	-2.0 [-9.9, 5.8]
At 2nd ANC visit^e^	8.6 [-18.9, 35.2]	9.5 [-17.2, 36.2]	10.2 [-16.1, 36.4]	25.3 [-2.1, 52.7]	26.7 [-0.4, 53.8]

Unless otherwise stated, reference group were children of women who were not infected at either 1^st^ ANC visit, 2^nd^ ANC visit or at delivery

ANC- Antenatal Care ref-reference category

*P-value <0.05

** *P*-value <0.01

*** *P*-value <0.001

^a^ Adjusted for maternal education, child sex and HOME score

^b^ Adjusted for maternal education, child sex, HOME score, preterm status and child weight-for-age

^c^ Adjusted for maternal education, gravidity, child sex, family possessions and HOME score

^d^ Adjusted for maternal education, gravidity, child sex, family possessions, HOME score, preterm status and child weight-for-age

^e^ Only one assessed child had a mother with malaria-helminth co-infection at 2^nd^ ANC visit.

After adjusting for gravidity, maternal education, family possession, child sex and HOME score, helminth infection at first ANC visit was negatively associated with infant gross motor function (p = 0.028). We observed that mothers who were infected with hookworms during the first ANC visit had children who scored less in the gross motor scale, -4.9 (95% CI: -8.6 to-1.1), compared to those whose mothers were never infected with hookworms at first ANC visit. With the exception of the association between gross motor scores and the occurrence of helminth infection over the course of pregnancy, sensitivity analyses performed by further adjusting for infant preterm status and weight-for-age, yielded similar results in the association between infants gross motor function and prenatal helminth infection. Helminth infection at second ANC was no longer statistically significantly associated with infant ELC scores after sensitivity analyses, p = 0.074 (see Table 5). Further adjustment for LBW (not preterm birth) and weight-for-age showed similar conclusions in the sensitivity analyses. We performed multiple regression analysis further adjusting for research nurses and found little difference in the results.

## Discussion

Our study has shown that intestinal helminth infection at first ANC visit is associated with poorer infant cognitive and gross motor functions at the age of one-year after adjusting for other known risk factors of cognitive and gross motor development. In our study population, prenatal hookworm infection was related to lower performance in gross motor tests. Our results also reveal that helminth infection at least once during pregnancy may have negative consequences on the cognitive and motor development of infants.

Our study is one of the few large prospective mother-child cohorts with relatively low attrition rate in francophone Africa[27] and including several assessments during pregnancy. To our knowledge, our study is the first to assess the impact of prenatal helminths on the psychomotor development of infants taking into account data from different stages of pregnancy. In addition, we used a comprehensive assessment for neurodevelopment carried out by research nurses specifically trained by an expert in cognitive assessment in African countries (co-author MJB). An additional strength of this study is the consideration of several potential confounding factors such as socio-economic status, maternal depression and RPM and HOME scores. Malaria has also been assessed several times during pregnancy allowing for the study of the impact of malaria-helminth co-infection on child development. Despite low power due to the low prevalence of co-infection, our results do not suggest a higher impact on child development of helminths when associated temporally with malaria. Maternal demographic and reproductive characteristics were also comparable between children lost to follow-up and those included in the study hence selection bias is unlikely.

Since pregnant women recruited in the trial had adequate antenatal care including at least, two ANC visits with treatment for helminth infection at first ANC visit (apart from emergency visits), our results are likely to underestimate the effect of prenatal helminth on infant cognitive function in the general population that may attend fewer ANC visits and receive fewer treatments. Also, the low sensitivity of the Kato-Katz technique for helminths[28] may have resulted in measurement error but since, in this prospective cohort, the assessment of helminth status was independent of the performance of infant in the MSEL at age 1 year, the misclassification would probably be non-differential of infant cognitive and motor scores thus the association may be biased towards the null. The low prevalence of *A*. *lumbricoides, T*. *trichiura* and *S*. *Mansoni* did not permit us to study their independent impact on infant cognitive function. Given that treatment was given to women after their first ANC visit, the number of chronic infections was low in our study. Therefore, the effect of chronicity of untreated prenatal helminth infection on child development could not be evaluated. By definition, helminth infection is chronic until treatment. Women testing positive for helminths may have been chronically infected prior to their first ANC visit. However, testing positive for helminth infection at second ANC visit and/or at delivery after being infected with helminths at first ANC does not specifically indicate chronicity. Instead it could indicate reinfection after being treated following mebendazole administration at first ANC visit. Our study is also limited in the inability to assess the presence of prenatal *S*. *haematobium* as urine samples were not examined for eggs of this species. Due to the low proportion of children assessed for helminth infection, we were not able to adjust for infants’ infections in models. Species of helminth in mothers and infants were largely different, yet regardless of the species there was no correlation between helminth infection in mothers and children. This therefore suggests that the association between maternal helminths and child development may be independent of infants’ helminths.

Apart from a cohort study that was nested within the Entebbe Mother and Baby Study in Uganda[19], we did not identify any published study on the impact of prenatal helminths on cognition in offspring. The negative relationship witnessed between maternal helminth infection and infant cognitive development in our study is consistent with the general conclusion in the aforementioned study. Converse to the findings of our studies, the authors found no association between maternal hookworm infection and infant neurocognitive development. One explanation may be that Nampijja et al.[19] excluded pregnant women presenting severe anemia (Hb concentration<80g/L). They also included some maternal and infant characteristics (such as maternal hemoglobin level and birth weight) in their final model. It is important to note that our study population had a low prevalence and a low intensity of helminth infection according to WHO classifications of the community endemic levels[22] and few cases of multiple infections with different helminth species.

The mechanism by which prenatal helminth infection influences infant cognitive function remains unknown. However, helminth infection especially with hookworms is a known risk factor for ID. When hookworms penetrate the intestinal mucosa of a host, they ingest the host’s blood causing intestinal blood loss and erythrocytes lysis[29]. This could result in IDA[30] which may be disadvantageous during pregnancy because of the increased physiological demand for iron. Studies have shown that in very iron deficient mothers, maternal serum ferritin concentration is correlated with that of the neonate[31] while decreased concentration is associated with a decrease in brain iron concentration[32] which could in turn alter hippocampal development of the neonate[33]. A study among one-year-old children found that, those born with inadequate brain iron stores (≤34μg/L cord ferritin) had lower psychomotor function and auditory recognition memory than those with adequate brain iron stores.[34]

Helminths may be associated with several adverse birth outcomes that could mediate the pathway between prenatal helminth infection and infant cognitive development. Although findings from clinical trials reveal no beneficial effect of anthelminthic treatment on LBW and preterm births[35], a large community study of about 5000 pregnancies in Nepal showed an increased risk of LBW and infant mortality among the children of women who did not receive antenatal anthelminthic treatment[36]. Notwithstanding the contradictory effects of prenatal helminth infection on birth outcomes, adverse birth outcomes have been confirmed by some studies to be associated with infant cognitive development.[37–39] Our results, after sensitivity analyses, however suggest that other plausible unmeasured factors could also account for the observed association between prenatal helminth infection and child development.

It is unlikely that increased susceptibility of children of infected mothers to helminth infection explains for the decreased ELC and gross motor scores, as there was no association between prenatal helminth infection and infant helminth infection by age one. Moreover, the pattern of helminth species varied in the mothers compared to the children. *T*. *trichuris* was the most prevalent species of helminths in children (20.9%) contrary to high hookworm prevalence in pregnant women.

Mebendazole is a broad-spectrum anthelminthic drug that is effective against several intestinal helminths. However, it has lower cure rates and fecal egg reduction rates for hookworms than albendazole.[40] In our study, although infection by any helminths at second ANC visits was not associated with poor cognitive or gross motor function, hookworm infection remained associated with ELC scores. This could be due to either the re-exposure of pregnant women to hookworms even after mebendazole administration or low cure rates against hookworms. Although we did not monitor the adherence to mebendazole treatment, the decline in parasite density at second ANC visit observed in the majority of pregnant women infected with the same species than at first ANC visit (see S1 Table) suggests good adherence.

### Conclusion

This study provides evidence of an association between intestinal helminths and hookworms among pregnant women and poor cognitive and gross motor functions in their children at approximately 12 months of age. In view of these findings and as recommended by the WHO, measures to prevent helminth infections should be reinforced. Further studies are needed to corroborate our findings and explain the pathophysiological mechanisms of this relationship.

## Supporting Information

S1 Table Parasite density among 10 women infected with hookworm at first and second ANC visit (DOCX)

S2 Table Data on cognitive function of children. (XLSX)
