# Supplementary material for: Impact of Helminth Infection during Pregnancy on Cognitive and Motor Functions of One-Year-Old Children
Source: PLoS Negl Trop Dis. 2015 Mar 10;9(3):e0003463. doi: 10.1371/journal.pntd.0003463 (PMC4355614; doi:10.1371/journal.pntd.0003463)
Supplement: S1 Table — (DOCX) [file pntd.0003463.s001.docx]

**Table S1**. Parasite density among 12 women infected with hookworm at first and second ANC visit

| Id | Hookworm density at 1^st^ ANC visit | Hookworm density at 2^nd^ ANC visit | Difference in hookworm density |
| --- | --- | --- | --- |
| 1 | 104 | 600 | 496 |
| 2 | 48 | 24 | -24 |
| 3 | 360 | 96 | -264 |
| 4 | 408 | 120 | -288 |
| 5 | 2736 | 600 | -2136 |
| 6 | 120 | 48 | -72 |
| 7 | 72 | 48 | -24 |
| 8 | 984 | 96 | -888 |
| 9 | 504 | 72 | -432 |
| 10 | 696 | 1416 | 720 |
| 11 | 576 | 72 | -504 |
| 12 | 24 | 24 | 0 |
